# Pilot study evaluating lipoma reduction with injected physiologic ice slurry

**DOI:** 10.3389/fvets.2025.1630506

**Published:** 2025-07-17

**Authors:** Kristen K. Arango, Cheryl A. London, William M. Karlin, Jacqueline Milton Hicks

**Affiliations:** ^1^Department of Clinical Sciences, Tufts University Cummings School of Veterinary Medicine, North Grafton, MA, United States; ^2^Department of Biostatistics, School of Public Health, Boston University, Boston, MA, United States

**Keywords:** canine, veterinary, lipolysis, lipoma, ice-slurry, coolant

## Abstract

**Objective:**

This study aimed to assess the adverse event profile following injection of canine lipomas with BXT-786 coolant, and to assess its impact on lipoma size.

**Methods:**

Ten healthy adult client-owned animals, each with two similarly sized, cytologically confirmed lipomas were enrolled. Lipomas were injected with either BXT-786 coolant or control room temperature 0.9% saline solution. Two blinded, independent observers measured lipomas with a caliper and ultrasound. Dogs were reevaluated at 2, 4, 8, and 12-weeks post-injections to assess for adverse events and repeat measurements. Lipomas were surgically removed at 12 weeks post injection and submitted for histopathologic analysis. Health related quality of life was assessed using an owner completed questionnaire. Response to treatment was determined and adverse events were reported and graded.

**Results:**

Based on caliper assessment, majority of the lipomas were determined to be stable in size prior to surgical excision after BXT-786 or saline injection. Greater variability in response assessment was observed when using ultrasound. No statistically significant change was found in patient quality of life over an 8-week period. Adverse events were typically unrelated to BXT-786 injection, although one dog did develop cellulitis and mild necrosis in the lipoma.

**Conclusion:**

Injection of BXT-786 coolant into lipomas was feasible and did induce some histopathologic changes consistent with effects on tumor cells, but it did not result in tumor shrinkage. Future studies could explore using coolants with more sustained coolant function and multiple injections to promote more efficient tumor reduction.

## 1 Introduction

Lipomas are slow-growing, benign fatty tumors that occur in multiple species, often occurring between skin and underlying muscle but also can be found within deeper tissues. The prevalence of lipomas in the canine population is estimated to be 12.47% (1). They are associated with increasing age and affected animals often develop multiple lipomas. Female dogs, those that are obese, and certain breeds are at an increased risk for this tumor (2). Lipomas are typically asymptomatic and readily diagnosed with fine needle aspiration and cytology (3). While most do not require treatment, some lipomas may elicit discomfort or become large enough to cause functional interference with normal activities (4). Surgical excision is typically curative. However, patient comorbidities and associated anesthetic risks along with costs may deter owners from pursuing removal.

To avoid surgical intervention, particularly for those dogs with multiple lipomas, alternative treatment strategies have been explored. Intralesional injection of a 10% calcium chloride solution was shown to induce lipoma regression, although it was associated with irritation and skin necrosis, thus limiting its clinical application (5). A retrospective study of 20 dogs with 76 simple, encapsuled lipomas demonstrated that dry liposuction was effective in removing lipomas in 96% of patients when the tumor diameter was less than 15 cm (6). However, regrowth was observed in 28% of dogs within 9–36 months post treatment (6). Lastly, ultrasound-guided intralesional injection of triamcinolone acetonide induced complete regression of 9 of 15 subcutaneous and subfascial lipomas (3). This was found to be relatively safe and effective although 6 dogs experienced polyuria/polydipsia for 2 weeks post treatment (3).

An alternative approach for addressing unwanted fat is cryolipolysis, a noninvasive method used for body contouring in human patients that employs principles of cooling to destroy adipocytes (7). As contacted tissues decrease in temperature, crystallization of lipids begins to occur, causing cell lysis and adipose tissue degeneration (7). Conventional cryolipolysis requires extracting heat from subcutaneous fat by conduction across the skin, thus limiting the depth and regional effectiveness of adipose tissue destruction (8). As such, standard cryolipolysis would not be effective for the typical dog lipoma. In the current study, a novel injectable coolant consisting of 15% glycerol in phosphate buffered saline, BXT-786 (Brixton Biosciences, Cambridge, MA, USA) was used to create an ice slurry that could be injected directly into the lipoma. This was previously demonstrated to induce subcutaneous fat loss in swine over several weeks following a single injection, with the amount of fat loss correlated to the volume of ice slurry injected (8). Importantly, there was no scarring or damage to surrounding tissue noted (8). Building upon this data, the primary objectives for this study were to assess the adverse event profile following injection of canine lipomas with BXT-786, and to assess its impact on lipoma size. We hypothesized that intralesional injection of the coolant would lead to effective cold induced lipolysis and therefore a non-surgical, minimally invasive treatment modality for lipoma reduction.

## 2 Materials and methods

### 2.1 Eligibility

The study protocol was approved by Tufts University’s Institutional Animal Care and Use Committee (G2021-152). Informed owner consent was required prior to enrollment. Eligibility criteria included dogs of any sex and breed, a minimum of 5 kg in body weight, and between the ages of 1 and 12 years of age with two cytologically confirmed similarly sized lipomas. Target tumor size was a minimum 2 cm and a maximum of 7 cm based on the longest diameter assessment. Additional criteria included a normal physical exam and lack of abnormalities on complete blood count and chemistry panel. Dogs were excluded from the study if they were pregnant, lactating or were deemed ineligible by investigators based on any significant liver, renal or cardiovascular disease.

### 2.2 BXT-786 preparation

BXT-786 is a proprietary mix of 0.9% saline and glycerol that permits the formation of an ice slurry amenable to injection through a large gauge needle. Custom-designed syringes containing room temperature, sterile BXT-786, were stored frozen at −22 to −18°C prior to preparation and injection. A custom-designed device (“dock”) was used for generating the BXT-786 coolant slurry immediately prior to injection.

### 2.3 Study design

Following screening and enrollment, two similarly sized lipomas on the same patient were designated A and B. Using a standard randomization table, these were assigned to be injected with either BXT-786 coolant or control room temperature 0.9% saline solution. Two blinded, independent observers measured the length, width and height of each lipoma twice, first with calipers and then using ultrasound (Toshiba Apilo 500, Toshiba Viamo PLT-1204 BT Transducer, San Jose, CA, USA) on Day 0. Observers performed their measurements separate from each other and were not present during the injection procedure. Dogs were sedated for injections with intravenous butorphanol alone (0.2 mg/kg), or in combination with dexmedetomidine (2 μg/kg); atipamezole of equal volume was used for intramuscular reversal in those cases. Lipoma sites were clipped and aseptically prepped prior to injection. BXT-786 slurry was administered at a maximum dose of 20 mL per lipoma based on the 7 cm size limit for the lipomas and the presumptive radius of cooling induced by this volume. A similar dose, to the fullest extent possible based on lipoma size, was used for the 0.9% saline injected into the control lipoma (Table 1). This volume was extrapolated from the previous swine study in which a total of 30 mL of BXT-786 was injected into the subcutaneous fat (8). A 17-gauge needle was used for both the BXT-786 slurry and saline injections.

**Table 1:** Volume of coolant and saline injected into paired patient lipomas.

Record ID	Coolant volume (ml)	Saline volume (ml)
Patient 1	20	8
Patient 2	14	14
Patient 3	20	20
Patient 4	18	18
Patient 5	15	15
Patient 6	20	14
Patient 7	20	20
Patient 8	17	17
Patient 9	20	15
Patient 10	10	10

Following the injection procedure, dogs were observed for 1 h to monitor for acute reactions. Dogs were reevaluated at 2, 4, 8, and 12-weeks post-injections to assess for adverse events and to repeat caliper and ultrasound measurements. At 2 weeks post injection, a complete blood count and chemistry profile were repeated. Both lipomas were surgically removed at 12 weeks post injections and submitted for histopathologic analysis. Health related quality of life was assessed via owner completed questionnaire at day 0 and week 8 using a previously established tool (9).

### 2.4 Response assessment

Response to treatment was assessed using the Veterinary Cooperative Oncology Group (VCOG) response evaluation criteria in solid tumors (RECIST v 1.1) (10). Two approaches were used. The first involved single observer baseline and final caliper or ultrasound longest diameter measurements for each tumor (A and B) using the following formula to determine percent change from baseline: [final length-baseline length]/baseline length. To account for variability in assessor measurement that might affect response assessment, the average baseline longest diameter measurement (caliper or ultrasound) across the two observers for each target lesion at baseline and at the final time point was used to determine a “final” response assessment: [final average length-baseline average length]/baseline average length. A complete response (CR) was defined as complete resolution of the tumor, either by measurement or as documented by histopathology (10). A partial response (PR) was defined as a ≥30% reduction in the target lesion, taking as a reference the baseline measurement (10). Progressive Disease (PD) was defined as a 20% or greater increase in the longest diameter of the target lesion, taking as a reference the baseline measurement (9). Stable Disease (SD) was defined as neither sufficient shrinkage to qualify as CR/PR, nor sufficient increase to qualify for PD (10).

### 2.5 Adverse events

Adverse events were reported and graded in accordance with the standard Veterinary Cooperative Oncology Group – Common Terminology Criteria for Adverse Events (VCOG-CTCAE) (11). Patients experiencing adverse events received appropriate medical therapy. Principal investigators graded the severity of adverse events and assigned attribution of the adverse event as “unrelated,” “unlikely,” “possible,” “probable” or “definite” in relation to coolant injection (11).

### 2.6 Statistical analysis

The reliability testing on tumor volume was performed by computing the concordance correlation coefficient (CCC) along with its 95% confidence interval. Reliability analyses were computed to estimate the reliability testing for repeated measures on tumor volume for ultrasound and caliper separately between the two observers and for the reliability testing for repeated measures on tumor volume irrespective of the measurement device used. A mixed effects model was used to compute the CCC, evaluating the observer effect on the change in tumor volume across visits, a subject specific random intercept was evaluated to account for the within-subject correlation due to repeated measurements using the *epiR* packages in RStudio (12). For the model that examines reliability testing irrespective of the measurement device used, a device measurement effect on the change in tumor volume across visit is included in the mixed effects model. A Bland–Altman plot was used to visualize the agreement with the limits of agreement (bias ± 1.96 std) calculated using Bland and Altman’s formula for repeated measures using the *BlandAltmanLeh* package in RStudio (13, 14). A paired t-test was used to assess changes in quality of life comparing baseline assessment to that at 8 weeks post injection.

## 3 Results

### 3.1 Demographics

Ten client-owned dogs were enrolled in the study following written owner consent. All medical histories and physical examinations obtained on enrolled dogs were clinically normal and revealed no overt signs of illness. Mean patient age was 10.18 years. The patient population consisted of eight castrated males and two spayed females. Breeds included mixed breed dogs (*n* = 5), Labrador Retrievers (*n* = 2) and one each of the following breeds: American Staffordshire Terrier, Belgian Malinois, and Cocker Spaniel.

### 3.2 Tumor injections

While every effort was made to identify dogs with similarly sized lipomas, this was not always possible. Consequently, the volume of injection of the 0.9% saline and the BXT-786 coolant was sometimes variable (Table 1, Case 1, 6, and 9). Additionally, due to the constraints of lipoma size, there were several instances where less than 20 mL total volume of coolant or saline was injected into the lipoma to prevent leakage of material out from the injection site secondary to increased interstitial pressures.

### 3.3 Response assessment

Response was assessed using RECIST criteria by measuring the longest diameter of each lipoma using calipers or ultrasound (Table 2). Based on caliper assessments, the majority of the tumors were determined to be stable in size prior to surgical excision by both observers. Discordant response assessments occurred for 2/10 of the placebo treated tumors and 3/10 of the BXT-786 injected tumors. However, based on the average response assessment across the two observers, 9/10 placebo and 7/10 BXT-786 were deemed to have achieved SD. Only two tumors were determined to have a PR by caliper assessment (one placebo, one BXT-786).

**Table 2:** Response assessment.

Patient #		Caliper				Ultrasound			
		Plac	Avg	BXT	Avg	Plac	Avg	BXT	Avg
1	Obs 1	SD	SD	SD	SD	N/A	–	N/A	–
	Obs 2	PR		PR		N/A		N/A	
2	Obs 1	PD	SD	PD	PD	PD	PD	PR	PD
	Obs 2	SD		SD		PD		PD	
3	Obs 1	SD	SD	SD	SD	SD	SD	SD	SD
	Obs 2	SD		SD		SD		SD	
4	Obs 1	PR	PR	SD	SD	SD	PD	SD	SD
	Obs 2	PR		SD		PD		SD	
5	Obs 1	SD	SD	SD	SD	SD	SD	PD	PD
	Obs 2	SD		SD		SD		PD	
6	Obs 1	SD	SD	PD	PD	PD	SD	SD	SD
	Obs 2	SD		PD		SD		SD	
7	Obs 1	SD	SD	SD	SD	SD	SD	SD	SD
	Obs 2	SD		SD		SD		PR	
8	Obs 1	SD	SD	PR	PR	SD	SD	SD	SD
	Obs 2	SD		SD		PD		SD	
9	Obs 1	SD	SD	SD	SD	PD	PD	SD	PD
	Obs 2	SD		SD		PD		PD	
10	Obs 1	SD	SD	SD	SD	SD	SD	PR	SD
	Obs 2	SD		SD		SD		SD	

SD = stable disease, PR = partial response, PD = progressive disease.

Baseline visit ultrasound longest diameter measurements are not available for patient 1 as the initial approach to ultrasound assessment using the Butterfly IQ+ device was found to be inadequate; the device was not suitable for capturing accurate length of the tumors due to limitations with the probe. Consequently, the Toshiba Apilo 300 system was used for all subsequent patients to provide more accurate assessments that could be readily stored in the Tufts PACS server for reference. Greater variability in response assessment was observed when using ultrasound as the measurement tool. In this setting, discordant response assessments occurred for 3/9 of the placebo treated tumors and 4/9 of the BXT-786 injected tumors. However, based on the average response assessment across the two observers, 6/9 placebo and 6/9 BXT-786 treated lipomas were deemed to have achieved SD using ultrasound. No tumors were determined to be PR by ultrasound. Importantly, no CRs were noted for any lipomas, regardless of the method used for assessment.

### 3.4 Adverse events and quality of life assessment

**Figure 1:**
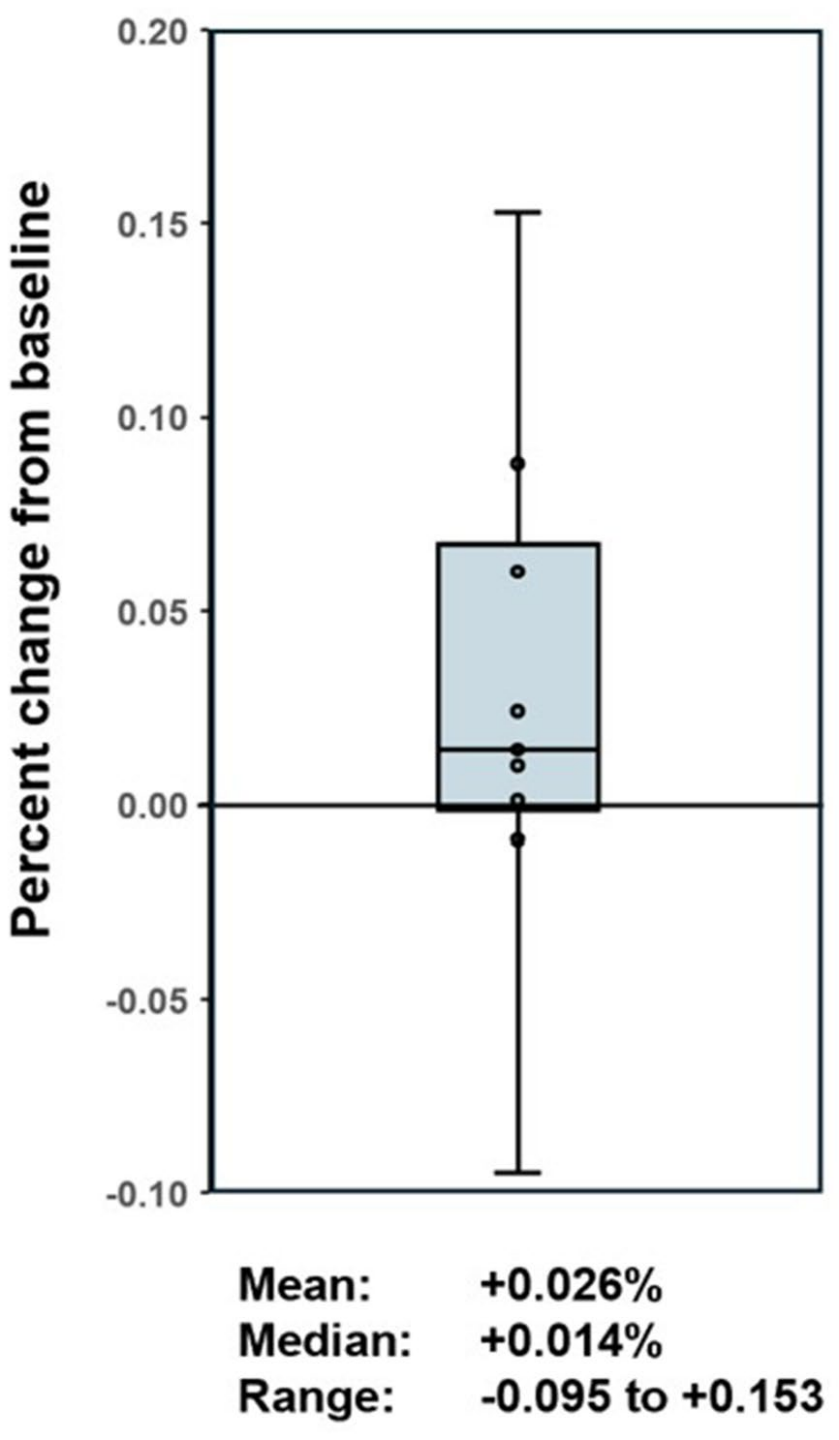
Box plot of health-related quality of life assessment score change from baseline.

A total of six adverse events were reported in four patients (Table 3), although most of these were deemed unrelated to the BXT-786 treatment. One dog developed cellulitis and infection at the coolant lipoma injection site necessitating early removal of the lipoma. Health related quality of life assessment aggregate data scores at baseline and week 8 post injection were compared. No statistically significant change was found over this time (95% CI −2.06, 7.3%, *p*-value 0.2405) indicating that the injections did not negatively impact quality of life (Figure 1).

**Table 3:** Adverse event profile.

Record ID	Adverse event	Grade	Attribution
Patient 1	Diarrhea	2	Unlikely
Patient 2	Pain on injection, vomiting, forelimb lameness	2, 2, 1	Possible, unlikely, unlikely
Patient 6	Cellulitis	3	Probable
Patient 7	Prolonged bradycardia	2	Unlikely

Veterinary Cooperative Oncology Group – Common Terminology Criteria for Adverse Events (VCOG-CTCAE) was used to grade reported adverse events and attribution determined by principal investigators.

### 3.5 Reliability and concordance of tumor assessment

Four measurements (ultrasound assessments of both lipomas by two independent observers, caliper measurements of both lipomas by two independent observers) were taken prior to treatment and at weeks 2, 4, 8, 12 of the study. Using the volumetric formula length × width × height = volume, the size of the tumor volume measured by ultrasound ranged from 1,297 to 76,319 mm^3^ with a mean of 2,030 mm^3^. The same tumors measured by caliper ranged from 3,240 to 223,015 mm^3^ with a mean of 41,284 mm^3^. This discrepancy is not unexpected given that calipers also include skin in the lipoma measurement.

**Figure 2:**
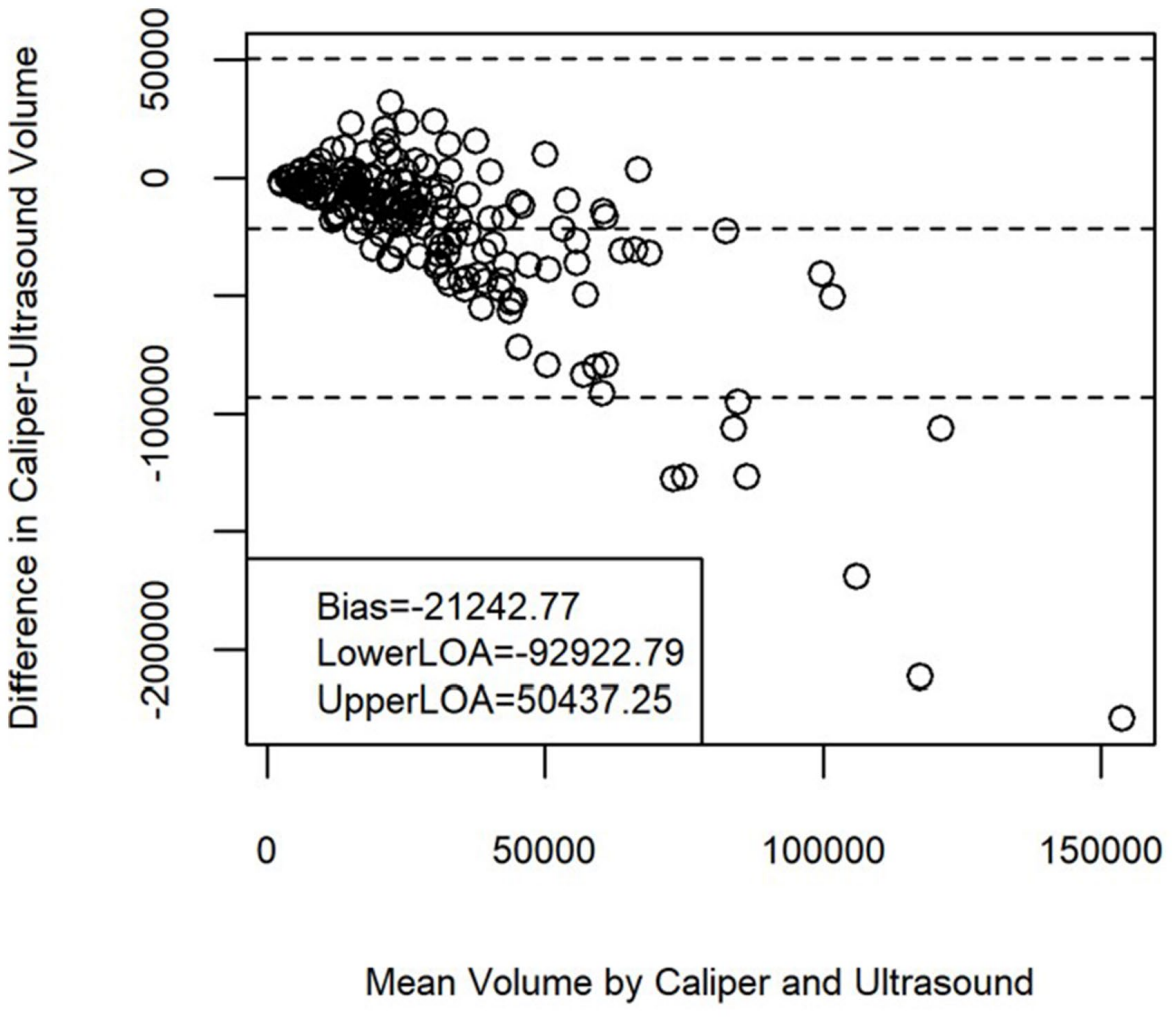
Bland–Altman plot of tumor volume for ultrasound versus caliper. The disagreement plot shows the difference between ultrasound and caliper methods against the average of the methods values for each subject. The two extreme lines are the +1 and −1 standard deviation.

Repeated measures of tumor size (volume calculations) were tested for reliability within each measurement modality. Relative concordance was achieved between observers for caliper (0.76, 95% CI: 0.66–0.83) while the concordance correlation coefficient was slightly larger for ultrasound (0.82, 95% CI: 0.74–0.88). Reliability testing for repeated measure on tumor volume irrespective of the measurement device measurement used, resulted in a relative concordance (0.79, 95% CI: 0.73–0.84). Concordance between ultrasound and caliper was also visually assessed using a Bland–Altman plot (Figure 2).

### 3.6 Histopathology

Histological evaluation of lipomas post excision demonstrated evidence of fibrosis, inflammation and necrosis in 8 of the 10 lipomas injected with BXT-786 coolant (Table 4). These changes are consistent with the proposed mechanism of action of the coolant. Conversely, inflammatory histopathologic changes were only reported in one lipoma injected with control room temperature sterile 0.9% saline. It is possible that in this instance, the large bore needle induced tissue injury that contributed to the observed changes.

**Table 4:** Histopathology results.

Record ID	Coolant lipoma	Control lipoma
Patient 1	Lipoma with extensive necrosis and reactive pyogranulomatous inflammation and fibrosis	Lipoma
Patient 2	Lipoma	Lipoma
Patient 3	Lipoma with regional granulomatous steatitis	Lipoma
Patient 4	Lipoma with granulomatous to pyogranulomatous inflammation	Lipoma
Patient 5	Lipoma with localized trauma, steatitis, fibrosis	Lipoma
Patient 6	Necrosis, granulation tissue and histiocytic inflammation, dermis subcutis and striated muscles	Lipoma, subcutis
Patient 7	Lipoma with necrosis, granulomatous inflammation and fibrosis	Lipoma
Patient 8	Lipoma with necrosis, granulomatous inflammation and fibrosis	Lipoma
Patient 9	Lipoma with focal fibrosis and mild edema/myoxomatous accumulation	Lipoma with multifocal granulomatous steatitis and fibroplasia
Patient 10	Lipoma, subcutis	Lipoma, subcutis

## 4 Discussion

This study aimed to assess the adverse event profile and for the induction of cold induced lipolysis following intralesional injection of the coolant BXT-786 into canine lipomas. The BXT-786 coolant leverages technology associated with the delivery of an ice slurry, which consists of a suspension of small ice particles in a carrier liquid of water (with or without solutes) that cause freezing point depression (8). As the ice particles melt, the injected slurry extracts a large amount of heat thereby delivering its thermal effect directly into target tissues. The prolonged cooling can then result in death of the affected tissues. A prior study undertaken in pigs showed a 55% (±6) reduction in adipose tissue thickness following injection of various biocompatible ice slurries compared with control sites (8). No adverse events from the treatment were noted, and there was no scarring or damage to surrounding tissue (8).

In our study, we did not see a significant biologic effect of BXT-786 when injected into canine lipomas. No complete responses to treatment were noted. RECIST based response assessment was generally consistent across observers when using calipers, with most lipomas exhibiting SD regardless of treatment group. Ultrasound based measurements demonstrated less concordance across observers, although again, most lipomas exhibited SD. It is commonly agreed upon that ultrasound examinations should not be used in clinical trials to measure tumor regression or progression because the examination is subjective and operator dependent (10). Additionally, entire examinations cannot be reproduced for independent review at a later date, and pressure applied may distort tumor size (10, 20). Evaluation of lesions by physical examination is also of limited reproducibility, but is permitted when lesions are superficial, at least 10 mm in size and can be assessed using calipers (10). Thus future studies should examine other measurement modalities, such as computed tomography (CT).

In the swine study, coolant was injected into subcutaneous adipose tissue and led to reduction in thickness and lobular panniculitis, similar to what is reported for cryolipolysis with topical cooling in humans (8, 15, 16). We did observe more histological evidence of inflammation and necrosis post coolant injection into canine lipomas when compared to their saline controls. Additionally, while BXT-786 did not result in effective cold induced lipolysis in most cases, our one severe adverse event was where a coolant injected lipoma became markedly inflamed and necrotic, requiring early removal at 4 weeks post injection. Principal investigators assigned attribution of this adverse event as probable or likely related to the coolant injection. As such, the histopathologic changes observed in the BXT-786 injected tumors are supportive of the proposed mechanism of action for cold induced lipolysis (Table 4).

A major difference between the study conducted in swine with BXT-786 and the current study is the targeted tissue. In the swine study, normal subcutaneous fat was injected, and this represents the typical anatomic location of human fat targeted in cosmetic cryolipolysis procedures. Lipomas are structurally and biologically different than normal fat tissue as they are technically benign tumors and typically encapsulated by a thin layer of fibrous tissues. Histopathologically, lipomas resemble surrounding normal fat but have larger cells, no nuclear hyperchromasia, and a delicate vascular network. It is therefore possible that lipomas may require a longer period of cooling/freezing to induce tumor cell death when compared to normal subcutaneous fat.

Relative concordance was found between measurements obtained through caliper and ultrasound, and between observers irrespective of modality used. The concordance correlation coefficient measures how well bivariate pairs of observations conform relative to a gold standard or another data set (17). It ranges from 0 to ±1, however disagreement in the strength assigned to the numeric values is present amongst researchers (17, 18). Altman suggested that it should be interpreted close to other correlation coefficients like Pearson’s, with <0.2 as poor and >0.8 as excellent (17). Consensus is that near ± 1 is perfect concordance (or discordance) and anything in-between should be interpreted with caution. The Bland Altman plot is a graphical method used to visually assess agreement between measurements (19). Our mean difference line is negative, indicating the presence of systematic bias, or that one measurement modality tends to underestimate tumor volume; the closer the mean difference is to zero, the better the agreement between the measures (19). Our data points are not randomly scattered around the mean bias line, and do not all fall within the upper/lower limits of agreement.

There were several additional limitations to this study. The first was the small sample size. Although adequate for a pilot study, a larger sample size would be necessary to make definitive conclusions regarding efficacy in clinical patients. It was challenging to identify and enroll patients with equal tumor size. Within our target tumor size range, 2–7 cm, we were unable to volume-match injections for patients 1, 6, and 9 (Table 1), and these volume differences may have led to the discrepancy in response by the independent assessors noted for patient 1 who had the largest volume difference. Despite this variable, there was no evidence that any of the BXT-786 injected tumors underwent significant reduction in size. We did not measure intratumoral temperatures to confirm that they were sufficiently low enough for a long enough period of time to induce tumor cell death. Additionally, in some of the tumors it was difficult to ascertain whether sufficient distribution of the coolant was achieved given the shape and size of the tumor. Dogs received only one injection of BXT-786, and while we did see evidence of change based on histopathologic evaluation, it is possible that multiple injections would be needed to cause actual tumor shrinkage.

In summary, while injection of the BXT-786 coolant into lipomas was feasible and did induce some histopathologic changes consistent with effects on the tumor cells, it did not result in substantial shrinkage of tumors, thus we reject our hypothesis. Future studies could explore using coolants with more sustained coolant function and multiple injections to promote more efficient tumor reduction.

## Data Availability

The original contributions presented in the study are included in the article/supplementary material, further inquiries can be directed to the corresponding author.
